# Fibrosis quantification in Hypertensive Heart Disease with LVH and Non-LVH: Findings from T1 mapping and Contrast-free Cardiac Diffusion-weighted imaging

**DOI:** 10.1038/s41598-017-00627-5

**Published:** 2017-04-03

**Authors:** Lian-Ming Wu, Rui Wu, Yang-Rongzheng Ou, Bing-Hua Chen, Qiu-Ying Yao, Qing Lu, Jiani Hu, Meng Jiang, Dong-Aolei An, Jian-Rong Xu

**Affiliations:** 1 Department of Radiology, Renji Hospital, School of Medicine, Shanghai Jiao Tong University, Shanghai, 200127 China; 2 Department of Radiology, wayne state university, Detroit, MI 48201 USA; 3 Department of Cardiology, Renji Hospital, School of Medicine, Shanghai Jiao Tong University, Shanghai, 200127 China

## Abstract

This study assessed the extent of fibrosis and the relationship between the ADC value and systolic strain in hypertensive patients with left ventricular hypertrophy (HTN LVH) and hypertensive patients without LVH (HTN non-LVH) using cardiac diffusion-weighted imaging and T1 mapping. T1 mapping was performed in 13 HTN LVH (mean age, 56.23 ± 3.30 years), 17 HTN non-LVH (mean age, 56.41 ± 2.78 years), and 12 normal control subjects (mean age, 55.67 ± 3.08 years) with 3.0 T MRI using cardiac diffusion-weighted imaging and T1 mapping. HTN LVH subjects had higher native T1 (1233.12 ± 79.01) compared with controls (1133.88 ± 27.40) (p < 0.05). HTN LVH subjects had higher ECV (0.28 ± 0.03) compared with HTN non-LVH subjects (0.26 ± 0.02) or controls (0.24 ± 0.03) (p < 0.05). HTN LVH subjects had higher ADC (2.23 ± 0.34) compared with HTN non-LVH subjects (1.88 ± 0.27) or controls (1.61 ± 0.38), (p < 0.05). Positive associations were noted between LVMI and ADC (Spearman = 0.450, p < 0.05) and between LVMI and ECV (Spearman = 0.181, p < 0.05). ADC was also related to an increase in ECV (R^2^ = 0.210). Increased levels of ADC were associated with reduced peak systolic and early diastolic circumferential strain rates across all subjects. Contrast-free DW-CMR is an alternative sequence to ECV for the evaluation of fibrosis extent in HTN LVH and HTN non-LVH, while native T1 has limited value.

## Introduction

Hypertension (HTN) is a common cardiovascular disorder and a primary cause of mortality and morbidity worldwide. Afterload and increasing arterial stiffness results in arterial hypertension, which leads to myocardium remodeling due to cardiomyocyte hypertrophy, fibroblast stimulation and increased collagen constitution. A progressive accumulation interstitial collagen fibers and left ventricular hypertrophy (LVH), which present with diffuse myocardial fibrosis, was demonstrated in necropsy examinations^1^ and endomyocardial biopsy^2–4^. LVH is a relatively independent high-risk factor to increase mortality and cardiac morbidity in certain HTN patients^5, 6^.

Hypertensive heart disease may exhibit different extents of fibrosis in different disease stages. Therapies specifically directed at hypertrophy and interstitial fibrosis may exhibit limited efficacy because of late therapy application (e.g., irreversible tissue-level changes after disease development)^7, 8^. Reliance on LV hypertrophy (LVH) and its regression to stratify risk has limitations^9, 10^. Therefore, evaluating cardiomyocyte hypertrophy non-invasively and tissue fibrosis in pathological LV remodeling specifically with therapies targeting these fundamental pathologies would be fundamental to pre-clinical drug evolution and the targeting of patients with the greatest potential benefit.

Recent cardiovascular magnetic resonance approaches to determine diffuse myocardial fibrosis involve a sequence of LGE imaging^11, 12^, post-contrast T1 mapping^13–15^ and ECV mapping^16, 17^. The latter two techniques provide quantitative measures (ECV values and T1) that further characterize the degree of fibrosis. However, contrast is required in these conventional techniques, and contrast is contraindicated in patients who suffer renal insufficiency. Contrast-free quantitative cardiovascular magnetic resonance techniques, such as pre-contrast T1 mapping^18^, diffusion imaging^19–21^, T1 ρ imaging^22^, and creatine chemical-exchange imaging^23^, have revealed myocardial fibrosis (i.e., scar) in chronic myocardial infarction patients. Pre-contrast T1 mapping^24^ and diffusion-weighted imaging^25, 26^ demonstrated extra value in the detection of diffuse myocardial fibrosis.

Consequently, the estimates of ADC may also be useful for the identification of diffuse and replacement fibrosis when an adequate extent of fibrosis appears (≥20%). We hypothesized that HTN LVH patients would exhibit diffuse myocardial fibrosis as evaluated by an ADC value compared to normotensive controls and HTN non-LVH. We also postulated that HTN LVH subjects would exhibit greater fibrosis, reduced systolic strain, and early diastolic strain rate compared to the other 2 groups.

## Methods

### Patients

Seventeen subjects with HTN ﻿non- LVH (mean age, 56.41 ± 2.78 years), 13 subjects with HTN LVH (56.23 ± 3.30 years), and 12 normotensive controls (mean age, 55.67 ± 3.08 years) were enrolled between November 2014 and October 2015. The Ethics Committee of Shanghai Renji Hospital approved all protocols, which were performed in accordance with approved guidelines. Informed consent was obtained from all subjects before the study began. Patients with a history of HTN and confirmed LVH using any imaging modality were considered for this study. Patients with any other causes of LVH, known coronary disease, significant valvular disease, patients with renal impairment with a glomerular filtration rate no greater than 45 ml/min/1.73 m^2^, or reduced systolic function (ejection fraction [EF] <45%) were excluded. Subjects with a history of HTN with diastolic blood pressure (DBP) greater than 90 mmHg or systolic blood pressure (SBP) greater than 140 mmHg on at least two office readings^27^ or who were taking 1 or more medications for hypertension were included. Subjects were classified as having LVH if their LV mass index (LVMI) using cardiac MRI was >81 g/m^2^ in men or >61 g/m^2^ in women as defined previously by Olivotto *et al*.^28^. Hypertensive subjects not meeting the criteria for LVH as defined in the preceding text were included in the HTN non-LVH group. Healthy volunteers who were normotensive with no history of HTN were enrolled in the control group.

### CMR protocol

Cardiac MR was performed using a 3.0-Tesla MR machine (Ingenia, Philips, Best, The Netherlands). A steady-state free precession sequence was used to evaluate LV mass and cardiac function analysis for cine imaging. The following sequence parameters were used: repetition time (TR) 2.8 ms, echo time (TE) 1.4 ms, flip angle 45°, field of view (FOV) (typically 300 × 300 mm^2^, but modified to minimize artifacts as needed and voxel size 1.2 × 1.2 × 7 mm^3^). Standard short-axis and long-axis orientations were obtained. A single breath-hold, steady-state free precession modified look-locker inversion recovery (MOLLI) technique^16, 29, 30^ used to generate T1 mapping. This protocol is a robust 5 s(3 s)3 s, which indicates at least 5 seconds for acquisition with images in 2 inversions, at least 3 seconds for recovery, and at least 3 seconds for acquisition with images in a second inversion. The starting TI (to the first image readout) was 100 ms with an increment of 80 ms for the subsequent look-locker trains. The pulse sequence parameters included: TE 1.1 ms, TR 2.5 ms, flip angle 35°, FOV 300 × 300 mm^2^, and voxel size 2 × 2 × 10 mm^3^. T1 mappings were obtained in basal and mid-ventricular short axis slices pre-contrast and 15 min after a bolus intravenous injection of 0.15 mmol/kg gadopentetate dimeglumine (Gd-DTPA) (Magnevist, Bayer Healthcare, Berlin, Germany). Hematocrit (Hct) was measured in all included subjects. LGE images were obtained approximately 10 minutes after bolus injection of 0.15 mmol/kg Gd-DTPA. A 2D Phase-Sensitive Inversion-Recovery sequence was used (TR 6.1 ms, TE 3 ms, flip angle 25°, FOV 300 × 300 mm^2^, and in-plane resolution 1.6 × 1.9 × 10 mm^3^). Diffusion encoding of the DW-CMR^31^ was performed during the most quiescent period of the cardiac cycle, which was identified using standard CINE imaging (end systole typically or end diastole), and mapping was phased to match the T1 and LGE breath-hold positions by the exhalation respiration. The following parameters of the compensated spin echo diffusion encoding sequence (one b0 image, three orthogonal diffusion directions, b = 350 s/mm^2^, second-order motion compensation diffusion-prepared bSSFP) were used: flip angle, 90°; repetition time msec/echo time msec, 4.1/2.0; bandwidth, 1449 Hz/pixel; echo train length, 61; 152 × 122 matrix; field of view, 230 × 230 mm; and voxel size, 1.2 × 1.2 mm^2^. The section thickness was 10 mm.

### Data analysis

Analyses were performed (Xu JR, Wu LM, who had more than 8 years and 3 years of history in cardiovascular radiology) using cvi42 (Circle Cardiovascular Imaging Inc., Calgary, Canada). End-systolic endocardial and end-diastolic and epicardial cavity regions were plane-metered for each slice of the short-axis. The LV mass, end-diastolic volume, and end-systolic volumes were determined and indexed to body surface area. LV mass was measured from the end-diastolic image frames using the validated CVI42. We included papillary muscles in measurements of LV mass as suggested in the recent Society for Cardiovascular Magnetic Resonance guidelines^15^. Cine imaging was also used to quantify circumferential strain and strain rate. Strain images were obtained in basal and mid short-axis locations. Peak systolic circumferential strain and early diastolic strain rates were measured. T1 maps were measured from the MOLLI data using a nonlinear least squares fit of the signal intensity versus T1 on a pixel-by-pixel basis, and the endocardial and epicardial borders of the myocardium were manually segmented using CVI42. The mean T1 in a region of interest in the ventricular cavity was taken as the blood T1. Diffusion encoding of the DWI sequence occurred at the most quiescent time of the cardiac cycle, which was confirmed using cine imaging (end systole typically or end diastole), and mapping was phased to match the T1 and LGE breath-hold positions by the exhalation respiration. The following parameters of the DW single-shot EP imaging sequence were used: flip angle, 90°; repetition time msec/echo time msec, 800/77; bandwidth, 1449 Hz/pixel; echo train length, 61; 152 × 122 matrix; FOV, 230 × 230 mm; and voxel size, 1.2 × 1.2 mm^2^. The section thickness was 10 mm.

### Statistical analysis

All continuous data are presented as the means ± standard deviation (SD). All statistical analyses were performed using SPSS version 18 (IBM Corporation, Armonk, New York). Continuous variables were analyzed using a two-sided Student’s non-parametric or t-tests when suitable. Statistics of variance (ANOVA) with Bonferroni correction were performed for multiple comparisons. Pearson’s correlation coefficient was used for correlation analyses for linear associations, and the Spearman’s rank correlation coefficient was used for monotonic nonlinear associations. A p value < 0.05 was regarded as statistically significant.

## Results

### Patient characteristics

Thirty-five HTN patients were primary involved in the analysis. The average age was 55 ± 5.56 years, and 63% were female. Five of the 35 HTN subjects were excluded because they did not meet the criteria for LVH as an LVMI that was greater than 61 g/m^2^ in women or 81 g/m^2^ in men as measured by CMR. A total of 30 patients were included in the study, and these subjects were further divided into 13 HTN LVH subject (56.23 ± 3.30 years) and 17 HTN non-LVH subject (mean age, 56.41 ± 2.78 years). Twelve normal volunteers (mean age, 55.67 ± 3.08 years) were also included in the study. Table 1 presents the basic characteristics of the 3 groups. The groups exhibited similar heart rate, LVEF, and age values. Left ventricular mass/volume ratios for the 3 groups were calculated. SPB and DBP were significantly higher in the HTN LVH group than the HTN non-LVH and control subjects. LV mass was higher in the HTN LVH group. Six subjects in the HTN non-LVH group had focal areas of late enhancement: 4 subjects had right ventricular insertion site hyper-enhancement, and the two subjects had a focal area of mid-wall enhancement in the distal inferior wall, which was non-ischemic in origin. These regions were not included on T1 and ADC mapping analyses to minimize potential bias from the inclusion of regions with focal fibrosis.

**Table 1:** Baseline Characteristics of HTN LVH, HTN non-LVH and Normotensive Controls.

Normotensive	HTN LVH (n = 13)	HTN non-LVH (n = 15)	Normotensive Controls (n = 12)
Sex	9 F; 4 M	11 F; 6 M	8 F; 4 M
Age (yrs)	56.23 ± 3.30	56.41 ± 2.78	55.67± 3.08
Systolic BP (mm Hg)	169.26 ± 11.32	163.32 ± 10.55	108.35 ± 8.42
Diastolic BP (mm Hg)	86.38 ± 5.63	81.33 ± 4.53	67.71 ± 4.94
Heart rate (beats/min)	73 ± 14	75 ± 12	71 ± 9
Number of HTN medicines	3.3 ± 1.4	2.4 ± 1.5	0
LVEF (%)	56.40 ± 3.85	57.21 ± 3.83	57.93 ± 2.92
LVMI (g/m2)	68 ± 4.56	73.35 ± 11.36	52.43 ± 10.91

### Native T1 value, Extracellular Volume and ADC

**Figure 1:**
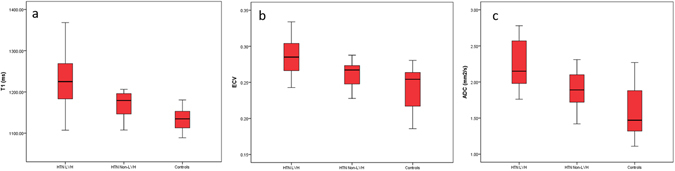
Box plot showing the distribution of Native T1 (**a**), ECV (**b**) and ADC values (**c**) in the 3 study groups. Boxes represent the 25th to 75th percentiles, and horizontal lines within the boxes represent the median values.

**Figure 2:**
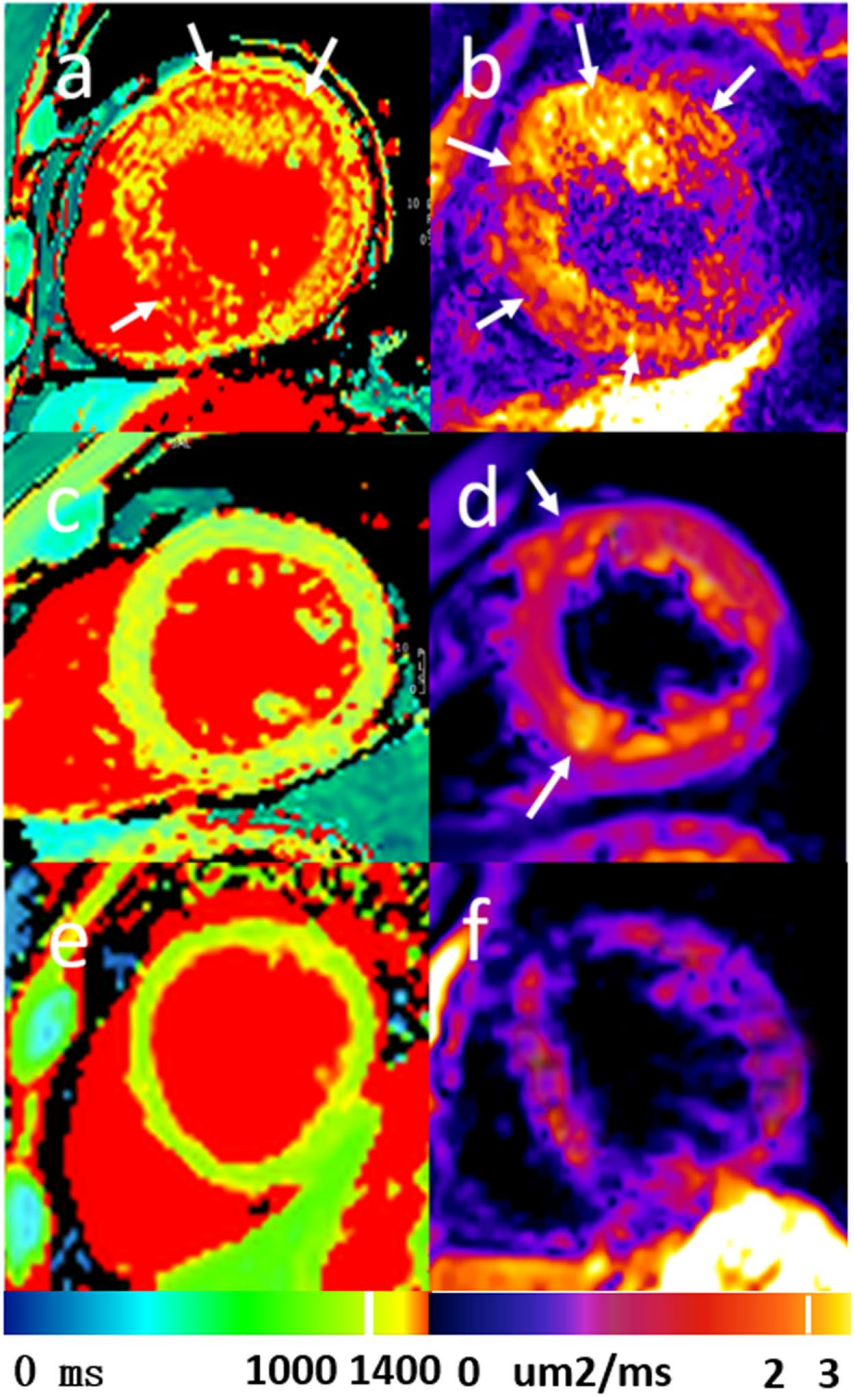
(**a**,**b**) Typical example of a 56-year-old male patient admitted with HTN LVH. Native T1 mapping showing diffuse fibrosis (white arrow) images (**a**), ADC mapping (**b**) showing diffuse fibrosis area, which was negative in the corresponding native T1 mapping (white arrow). (**c**,**d**) Typical example of a 55-year-old male patient admitted with HTN non-LVH. Native T1 mapping (**c**) showing negative fibrosis area and ADC mapping (**d**) showing focal fibrosis (white arrow), which was negative in native T1 mapping. (**d**,**e**) Typical example of a 36-year-old normal control. Native T1 mapping (**d**) and ADC mapping (**e**).

The T1 values were significantly higher in the HTN LVH subjects versus controls (1233.12 ± 79.01 versus 1133.88 ± 27.40, p < 0.05). No significantly differences were found in T1 values between HTN LVH and HTN non-LVH (1233.12 ± 79.01 and 1168.23 ± 33.61, p > 0.05). No significant differences were found in T1 values between HTN non-LVH and normal controls (1168.23 ± 33.61 and 1133.88 ± 27.40, respectively, p > 0.05). ECV values were greater in HTN LVH subjects versus the normal control group (0.28 ± 0.03 versus 0.24 ± 0.03, p < 0.05) and HTN non-LVH subjects (0.26 ± 0.02, p < 0.05). There were no significant differences in ECV values between normal control and HTN non-LVH subjects. ADC values were significantly higher in HTN LVH subjects versus controls (2.23 ± 0.34 vs. 1.61 ± 0.38, p < 0.05) and HTN non-LVH (1.88 ± 0.27, p < 0.05). There were no differences in ADC values between normal control subjects and HTN non-LVH subjects (Table 2, Fig. 1). Figure 2 shows typical examples of HTN LVH, HTN non-LVH and normal subjects.

**Table 2:** Subgroups analysis.

	HTN LVH (n = 13)	HTN non-LVH (n = 17)	Normotensive Controls (n = 12)
ECV	0.28 ± 0.03	0.26 ± 0.02	0.24 ± 0.03^†,*^
Native T1 (ms)	1233.12 ± 79.01	1168.23 ± 33.61	1133.88 ± 27.40*
ADC value (﻿mm^2^/s﻿)	2.23 ± 0.34	1.88 ± 0.27	1.61 ± 0.38^†,*^

Values are mean ± SD. *p < 0.05 vs. HTN LVH. ^†^<0.05 vs. HTN non-LVH; ECV = extracellular volume; HTN = hypertensive; LVH = left ventricular hypertrophy. Extracellular volume, Native T1, and Native T1 Values in HTN LVH, HTN non-LVH and Normotensive Controls.

### Relationships among ECV, ADC and LVMI

**Figure 3:**
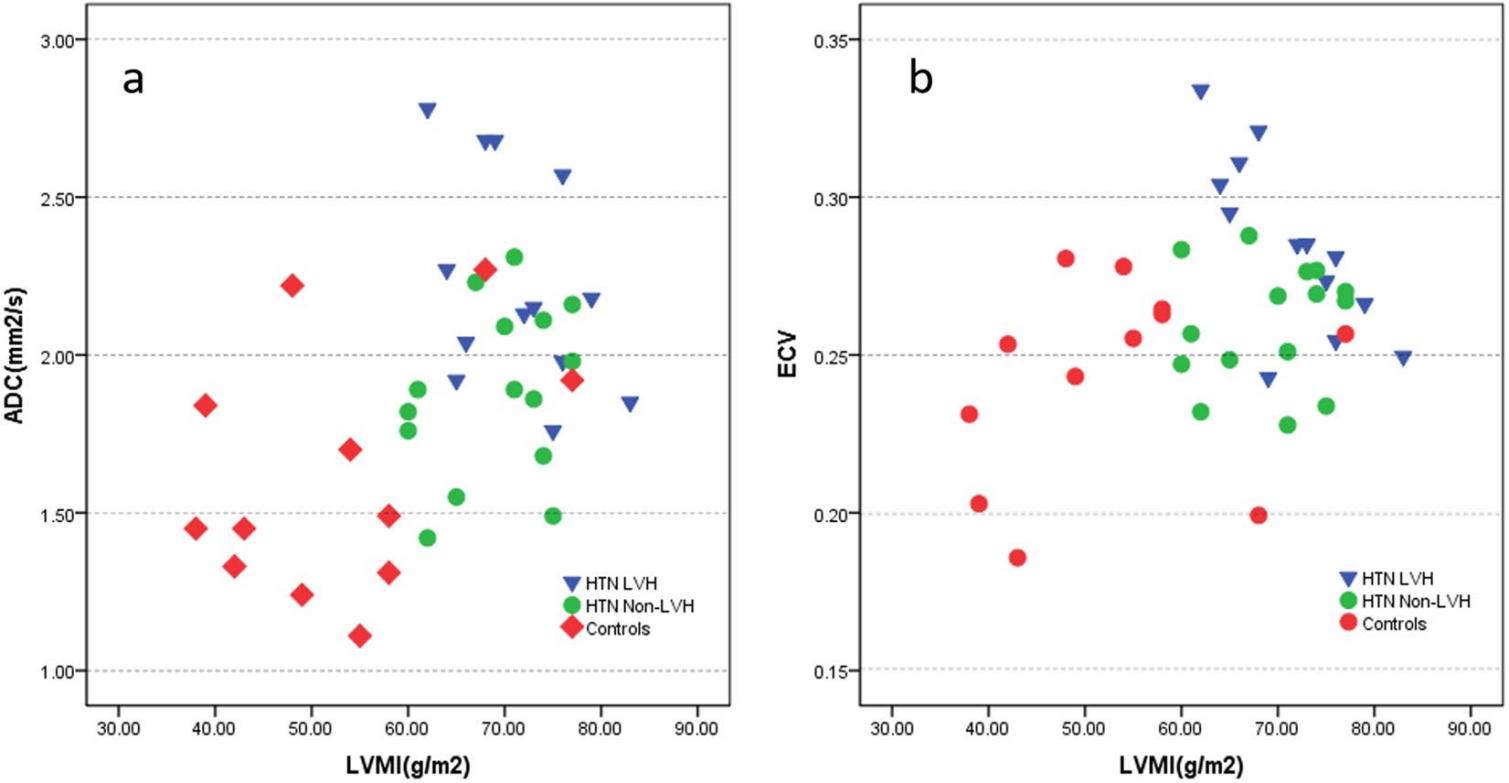
(**a**) Spearman correlation shows a positive association (Spearman rho = 0.26, p < 0.05) between ADC and LVMI. (**b**) Spearman correlation shows a positive association (Spearman rho = 0.18, p < 0.05) between ECV and LVMI.

**Figure 4:**
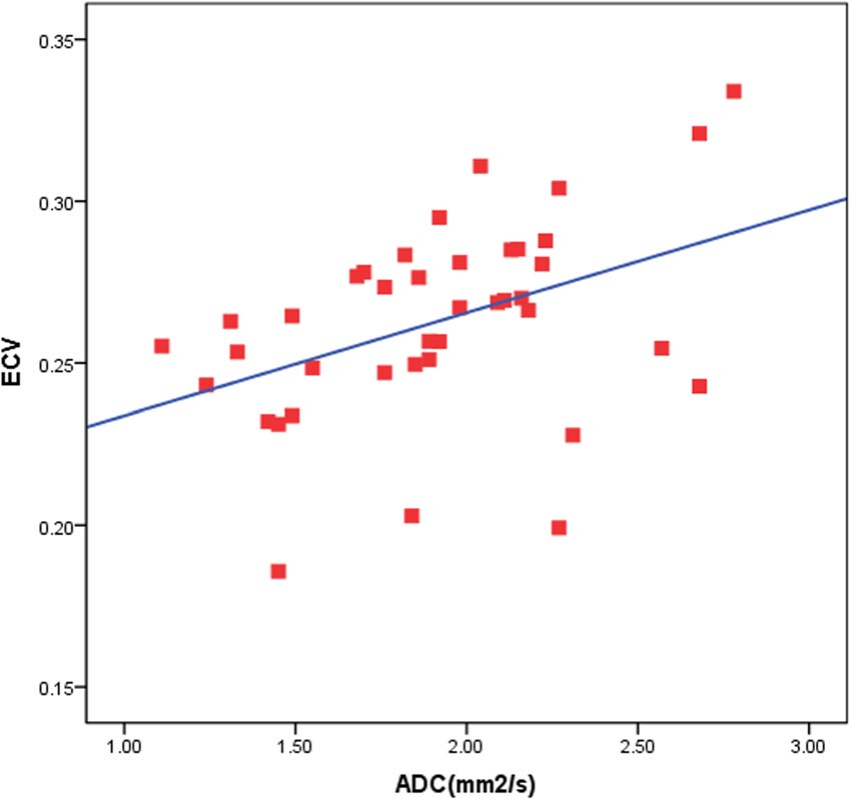
(**a**) ECV versus ADC. Pearson’s correlation shows a linear association between ADC and ECV (R = 0.1, p < 0.05).

Positive associations were noted between LVMI and ADC (Spearman = 0.450, p < 0.05) (Fig. 3a) and between LVMI and ECV (Spearman = 0.181, p < 0.05) (Fig. 3b). However, ADC and ECV were not linearly related to an increase in LVMI. ADC was also related to an increase in ECV (R^2^ = 0.210) (Fig. 4). HTN non-LVH subjects had similar ADC and ECV levels as controls. HTN LVH subjects had greater LVMI levels and higher ADC, ECV levels. However, subjects with the highest LVMI did not show a proportional increase in ADC and ECV, and certain HTN LVH subjects, with relatively lower LVMI, exhibited significantly higher levels of ADC and ECV.

### ADC, circumferential strain, and strain rate

**Figure 5:**
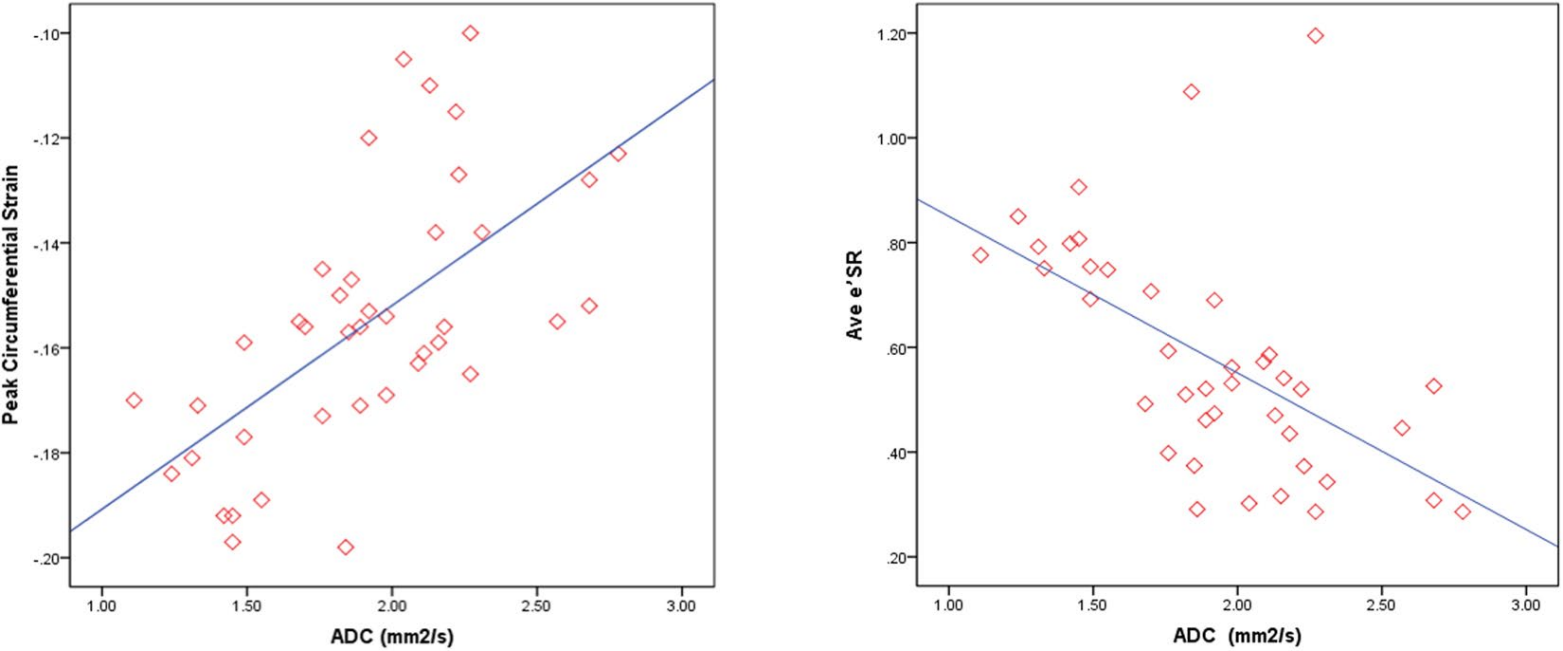
(**a**) Peak circumferential strain versus ADC. Pearson’s correlation shows a linear association between ADC and peak circumferential strain (R = 0.392, p < 0.05). (**b**) Mean early diastolic circumferential strain rate versus ADC. Pearson’s correlation shows a linear association between ADC and peak circumferential strain rate (R = −0.305, p < 0.01).

Peak circumferential strains at basal and mid-ventricular levels were reduced in all HTN LVH subjects compared to normotensive control subjects (Table 3). ADC correlated with peak circumferential strain (R = 0.392, p < 0.05) (Fig. 5a). The mean early diastolic circumferential strain rate (e’SR) was also reduced in HTN LVH subjects compared to HTN non-LVH and normal control subjects (Table 3). The results were similar for early diastolic circumferential strain rates at basal and mid-ventricular levels (Table 3). ADC correlated significantly with the mean early diastolic circumferential strain rate (R = −0.305, p < 0.01) (Fig. 5b).

**Table 3:** Strain and Strain Rate among the HTN LVH, HTN non-LVH and Normotensive Controls.

	HTN LVH (n = 13)	HTN non-LVH (n = 17)	Normotensive Controls (n = 12)
Peak base circumferential strain	−0.12 ± 0.02	−0.16 ± 0.02	−0.17 ± 0.02*
Peak mid-circumferential strain	−0.12 ± 0.02	−0.15 ± 0.02	−0.17 ± 0.02*
Peak average circumferential strain	−0.12 ± 0.02	−0.15 ± 0.02	−0.17 ± 0.02*
Base e’SR (s^−1^)	0.41 ± 0.11	0.53 ± 0.13^#^	0.83 ± 0.19*
Mid e’SR (s^−1^)	0.37 ± 0.12	0.53 ± 0.12^#^	0.82 ± 0.15*
Average e’SR (s^−1^)	0.39 ± 0.10	0.53 ± 0.12^#^	0.83 ± 0.17*

Values are Mean ± SD. *p < 0.05 vs. HTN LVH, ^#^p < 0.05 vs. HTN non-LVH. e’SR = early diastolic circumferential strain rate; LVH = Left ventricular hypertrophy.

## Discussion

This study demonstrated that T1 mapping and DWI using CMR revealed greater diffuse fibrosis as measured by ECV and ADC values in subjects with HTN LVH compared to HTN non-LVH and control subjects. Higher ADC values were associated with reduced peak systolic circumferential strain and early diastolic circumferential strain rate across all subjects. Native T1 levels were longer in HTN LVH subjects compared to normotensive controls. ADC, ECV and native T1 values exhibit different sensitivities to underlying changes in the intracellular and extracellular spaces within the myocardium. Native T1 is a function of the relaxation of water in the extracellular and intracellular compartments, and it may reflect changes in cellular hypertrophy and extracellular fibrosis. There is a fast water exchange between these compartments, and the contribution of the intracellular and extracellular compartments cannot be separated without contrast administration. Gadolinium contrast agents accumulate solely in the extracellular space. Therefore, the post-contrast T1 shortening is primarily due to a shortening of T1 relaxation time in the extracellular space (although the measured T1 is affected by water exchange with the intracellular compartment)^32^. The ECV is calculated from a combination of native and post-contrast calculations and reveals the volume fraction of extracellular space on a pixel-wise basis. Therefore, native T1 and ECV are sensitive to different compartments within the myocardium and may each reflect different aspects of the pathological changes of hypertensive heart disease. DWI interprets the underlying changes in the extracellular and intracellular spaces of the myocardium. A low b value of DWI may reflect the extracellular compartments, which records the changes in extracellular fibrosis. This study clinically disclosed the ability of DWI to serve as an alternative contrast-free sequence to ECV and LGE for diffuse fibrosis assessment. The present study extends previous research of the ability of DWI to observe replacement fibrosis^20, 25, 26^ and illustrated an extra value to visualize diffuse myocardial fibrosis presentations compared to LGE and ECV. Our preliminary experience using DWI in HTN patients supports the use of DW-CMR to distinguish different extents of fibrosis.

Myocardial fibrosis is a usual end point of numerous cellular and non-cellular pathological processes in hypertensive heart disease. The pathogenesis of hypertensive heart disease involves myocardium remodeling within the perivascular space, muscle fibrosis, medial hypertrophy of the intra-myocardial coronary vasculature, and cardiomyocyte hypertrophy^33^. The mechanisms of the evolution and progression of different LVH patterns are not completely defined but involve the duration and severity of hypertension, the effects of cytokines and neurohormones, development factors, and hereditary predisposition^34^. Myocardial fibrosis leads to systolic and diastolic disorders, myocardial ischemia, and ventricular and atrial arrhythmias^35^. A solid correlation was demonstrated between increased myocardial collagen content and left ventricular diastolic disturbance using echocardiography^36^. This study also demonstrated that losartan treatment decreased the left ventricular stiffness and collagen content. This result suggests that the diffuse fibrosis observed in hypertensive patients is reversible using certain antihypertensive therapies. Reduction in diffuse fibrosis may improve diastolic function, systolic function, and outcomes. Therefore, measurement of diffuse fibrosis levels may serve as a therapeutic target of the efficacy of antihypertensive and other therapies.

The present study demonstrated that HTN LVH subjects exhibited decreased peak systolic and significant e’SR and circumferential strain compared to normal control subjects. Our results are consistent with a previous study^37^. The current research enhanced previous results because it examined the behavior of circumferential myocardial mechanics in relation to the extent of fibrosis and hypertrophy. The three subgroups (HTN LVH, HTN non-LVH and normal control) exhibited different degrees of replacement fibrosis, interstitial fibrosis and hypertrophy. We further revealed a positive correlation among the peak systolic circumferential strain, average e’SR, and increasing levels of diffuse fibrosis as measured using ADC, which is consistent with the expected relationship between myocardial function and fibrosis. One possible mechanism linking ADC to reduced systolic strain in HTN LVH patients may be increased extracellular matrix deposition, which leads to increased LV stiffness and results in reduced end diastolic muscle fiber length, cardiac muscle contraction and LV systolic strain. Previous studies demonstrated that diffuse fibrosis was associated with worsening systolic/diastolic function and adverse LV remodeling^35^. However, further larger multi-center studies are required to verify this hypothesis. Unlike other groups have found higher correlations, ADC vs ECV correlations were quite low. As we know varying amounts of fibrosis were frequently seen in hypertrophic cardiomyopathy (HCM), and can be detected by T1 mapping. But in some cases myocardial injuries also may be found in patients with HCM^38, 39^. We guess the reason why other groups have found higher correlations than ADC vs ECV is that the myocardial injuries may influence the excellular volume.

Conventional cardiac DWI sequence is highly sensitive to bulk motion, which may cause some false-positive results. It is also sensitive to some artifacts, especially artifacts associated with geometric distortions due to magnetic susceptibility differences across the imaging of different tissues and bulk motions. The ultimate signal intensity may be an invalid estimate for the mentioned artifacts. The magnetic susceptibility may be addressed using parallel imaging with zonal excitation. The DWI sequence used in the present study was compensated spin echo diffusion encoding. This sequence was used in several studies^31, 40–42^, and it is a feasible method to overcome bulk motion. We used compensated spin echo diffusion encoding to ensure that the sequence was sufficiently insensitive to systolic bulk motions of the heart. Nguyen *et al*.^43^ also used DWI technique to detect diffuse myocardial fibrosis in hypertrophic cardiomyopathy patients and compare its performance with established T1 mapping. They reported that DWI is sensitive to determine diffuse myocardial fibrosis and is capable of characterizing the extent of fibrosis in HCM patients. The main outcome is similar as ours, the present study found DW-CMR is an alternative sequence to ECV for the evaluation of fibrosis extent in HTN patience. However, the presented work demonstrated several additional findings. The subgroup analysis of the ADC measurement for HTN LVH and HTN non-LVH patients confirmed the DWI was capable of characterizing the extent of fibrosis in HTN patient. Furthermore, the relationship between ADC and systolic strain in HTN patient was also analyzed. We noted that higher ADC values were associated with reduced peak systolic circumferential strain and early diastolic circumferential strain rate across all subjects. Which was consistent with the expected relationship between myocardial function and fibrosis.

Unlike conventional T1 MOLLI sequences, such as the 3(3)3(3)5 protocol, the sequence used in our research was a robust 5 s(3 s)3 s, which indicates that there were at least 5 seconds for the acquisition of images in 2 inversions, at least 3 seconds for a recovery, and at least 3 seconds for acquisition of images in a second inversion. Our MOLLI approach used “seconds” instead of “beats”. Therefore, the scan time was virtually independent from the heart rate. This method is unlike schemes that use “beats”, in which scan time depends strongly on heart rate. A new scheme is preferable over the original 3-3-5 scheme (3b(3b)3b(3b)5b) because of the significantly shorter breath-hold scan time and increased accuracy. The effect of variable heartbeats on T1 values may be excluded by using the new T1 MOLLI sequence in our research. The large spread of the HTN LVH group compared to the HTN non-LVH and controls may be attributed to the extent of myocardial fibrosis.

The small sample size of the total and subset categories is the first limitation of the study, The effect of variables, such as age and gender, could not be assessed. A larger multicenter study is required to confirm our results for widespread clinical use. Secondly, there are circumstances in which good quality T1-maps are not obtainable. We could not perform native T1-mapping in 3 patients in our study because of significant arrhythmia. There are different methods of T1 mapping, and further research is needed to establish a standardized method of T1 mapping for clinical use, despite the recently published consensus guidelines for the use of T1 mapping. Our method used the standard MOLLI technique and produced similar results. Thirdly, the DWI method in the current study was based on the artificial result of the most quiescent period, which may generate diffusion preparation compensated by motion. Discrete triggering and/or the duration of end diastole was substantially shortened by the high and unstable heart rates of the subjects. Therefore, approximately half of the subjects required end systolic triggering. The automatic or semi-automatic techniques are more likely to be push-button techniques because of the triggering determinants of the ideal cardiac phase. Another predominantly realistic fault of the DWI method in the current research was the relative low spatial coverage (8-mm thickness x 4 slices with 32-mm coverage). Entire coverage (~80 mm) was realized using this DW-CMR method, but it required no less than 12.5 min of scan time. HTN subjects with greater LV masses are required to have a minimum scan of approximately 15 min to contain the entire LV (~100 mm) adequately. Consequently, the overall research design is limited by this technique. An estimate of the whole LV fibrosis load could not be assessed in the general research design.

## Conclusion

Contrast-free non-invasive quantitative DWI is a feasible alternative to the native T1 value and established contrast-enhanced ECV-CMR for the identification of diffuse myocardial fibrosis in HTN patients. HTN LVH patients exhibited greater diffuse fibrosis and reduced circumferential strain and circumferential strain rates compared to HTN non-LVH and control subjects. Diffuse fibrosis is linearly related to a worsening of circumferential strain, but variations in ADC among patients with LVH may provide insight into the differential expression of fibrosis and myocyte hypertrophy among patients with HTN. ADC measurement may be a useful novel target to monitor the efficacy of therapies for HTN patients.

## Electronic supplementary material

Supplementary Figure Legends
