# Supplementary material for: Fibrosis quantification in Hypertensive Heart Disease with LVH and Non-LVH: Findings from T1 mapping and Contrast-free Cardiac Diffusion-weighted imaging
Source: Sci Rep. 2017 Apr 3;7:559. doi: 10.1038/s41598-017-00627-5 (PMC5428770; doi:10.1038/s41598-017-00627-5)
Supplement: Supplementary file 1 — Supplementary Figure Legends [file 41598_2017_627_MOESM1_ESM.pdf]

Title:

Fibrosis quantification in Hypertensive Heart Disease with LVH and Non-LVH：Findings from T1 mapping and Contrast-free Cardiac Diffusion weighted imaging.

Authors:

Lian-Ming Wu [1, #], Rui Wu[1, #]， Yang-Rongzheng Ou[1], Bing-Hua Chen[1]， Qiu-Ying Yao[1]， Qing Lu[1] , Jiani Hu[2], Meng Jiang[3], Dong-Aolei An[1],Jian-Rong Xu[1].

Supplementary Figure

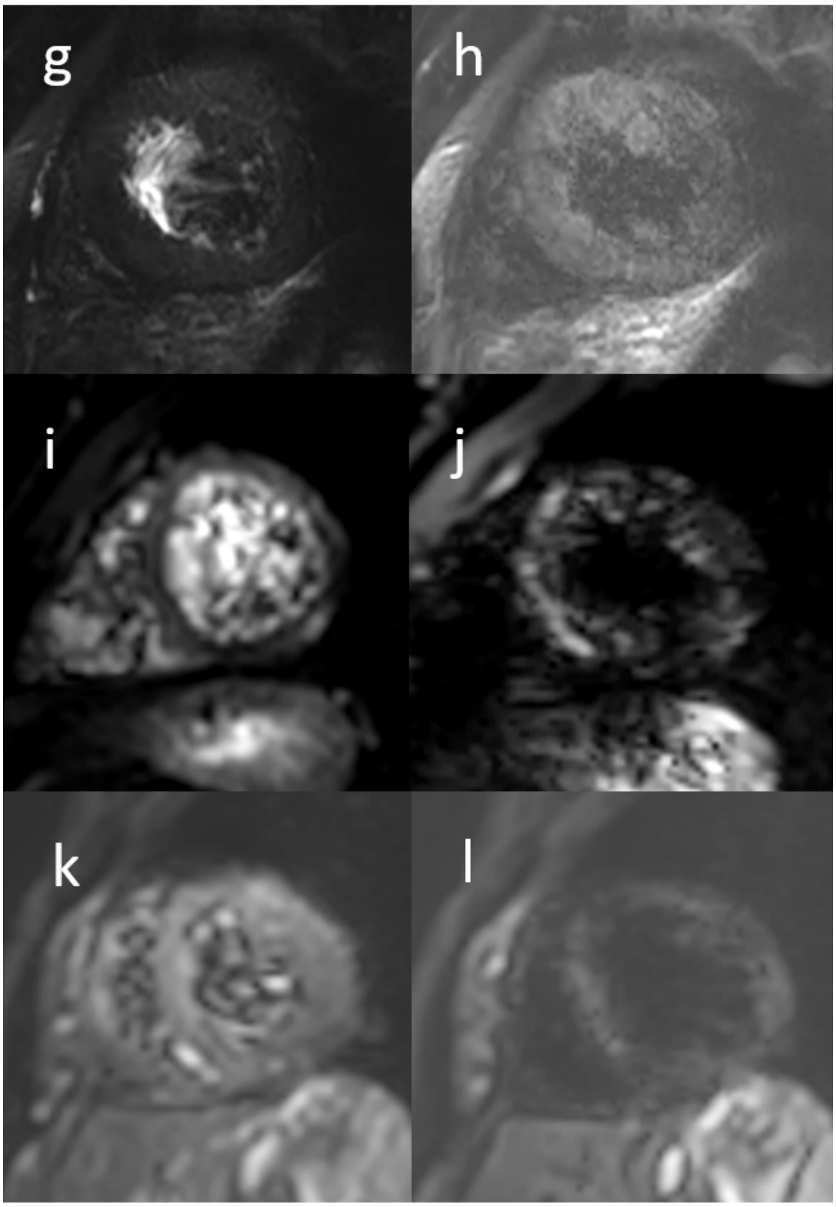

Supplementary Figure Legends

Supplementary Figure

. (g-h) Typical example of a 56-year-old male patient admitted with HTN LVH. Raw diffusion weighted images b=0 s/mm2 (g), b=350 s/mm^2 (h). (i-j) Typical example of a 55-year-old male patient admitted with HTN non-LVH. Raw diffusion weighted images b=0 s/mm^2 (i), b=350 s/mm^2 (j). (k-l) Typical example of a 36-year-old normal control. Raw diffusion weighted images b=0 s/mm^2 (k), b=350 s/mm^2 (l).
